# Automated Renal Tumor Segmentation in Computed Tomography Images Using a Global Attention–Based DeepLabV3+ Model: Algorithm Development and Validation

**DOI:** 10.2196/78523

**Published:** 2026-09-15

**Authors:** Yueyan Zhao, Jianqiang Liu, Lingyu Shao, Lin Li, Zhaoqing Liu, Yujie Liu, Jiaxin Wen, Xinyao Hao, Shuyan Li, Jianhong Zhao, Boming Song

**Affiliations:** 1 School of Medical Information and Engineering, Xuzhou Medical University, KJL_E202 Room, 209 Tongshan Road, Yunlong District, Xuzhou, Jiangsu, 221004, China, 86 15852036109; 2 Department of Radiology, The Second Hospital & Clinical Medical School, Lanzhou University, Lanzhou, Gansu, China

**Keywords:** kidney tumor segmentation, CT images, DeepLabV3+, global attention mechanism, deep learning, KiTS19

## Abstract

**Background:**

The rising global incidence of renal tumors necessitates precise diagnostic interventions. Accurate segmentation of computed tomography (CT) scans is essential for nephron-sparing surgery and radiotherapy. However, conventional manual delineation is labor-intensive and prone to significant interobserver variability due to tumor morphological heterogeneity. There is an urgent clinical demand for robust, automated segmentation solutions.

**Objective:**

This study aims to develop and validate GAM-DeepLabV3+, an automated framework designed to address boundary ambiguity and high false-positive rates in complex renal imaging scenarios.

**Methods:**

We propose an optimized encoder-decoder architecture specifically tailored for renal mass detection. The framework incorporates three key innovations: (1) a lightweight MobileNetV2 backbone to minimize computational overhead for clinical deployment; (2) an Atrous Spatial Pyramid Pooling (ASPP) module to capture multiscale contextual information; and (3) a Global Attention Mechanism (GAM) in the decoder to enhance channel-spatial interactions, thereby refining boundary delineation by suppressing background noise. The model was rigorously evaluated on a private clinical dataset (n=218) and the KiTS19 benchmark (n=210).

**Results:**

GAM-DeepLabV3+ consistently outperformed state-of-the-art baselines. On the private dataset, the model achieved a mean Dice similarity coefficient (DSC) of 0.939 (SD 0.008), significantly surpassing feature pyramid network (FPN; mean 0.893, SD 0.008; *P*<.001) and no-new-Net (nnU-Net; 2D, custom; mean 0.908, SD 0.013; *P*<.001). It also achieved a mean 95% Hausdorff distance (HD95) of 1.485 (SD 0.522) pixels. On the KiTS19 dataset, it maintained a mean robust DSC of 0.928 (SD 0.006). To facilitate clinical translation, a demonstration-only online platform was developed.

**Conclusions:**

The GAM-DeepLabV3+ framework provides an accurate, efficient, and fully automated solution for renal tumor segmentation. By overcoming boundary ambiguity and optimizing feature fusion, this approach shows potential as a decision-support aid, pending future validation with 3D reconstruction.

## Introduction

The kidney is a parenchymal organ situated bilaterally along the spinal column beneath the costal arches, encapsulated by a thin layer of connective tissue and adipose tissue. It plays essential physiological roles in excreting metabolic waste products, maintaining water-electrolyte homeostasis, and secreting critical hormones, including erythropoietin and renin. Against the backdrop of a continuously rising global burden of malignant disease, kidney tumors [1,2] have emerged as one of the most prevalent malignancies of the urinary system. Epidemiologically, their incidence ranks second only to bladder cancer among urological tumors. In 2020 alone, more than 430,000 new cases of kidney cancer were diagnosed worldwide, and the incidence continues to rise with advances in diagnostic technology and accelerating population aging, posing formidable challenges to kidney cancer prevention and treatment across diverse health care settings [3]. The World Health Organization (WHO) has formally designated kidney tumors as a critical global public health priority [4].

Kidney tumors encompass 3 principal histological categories [5]: clear cell renal cell carcinoma (ccRCC), Wilms tumor, and transitional cell carcinoma of the renal pelvis. ccRCC, originating from the proximal tubular epithelium of the kidney, constitutes the most common primary renal malignancy in adults, accounting for approximately 80%‐85% of all cases. Wilms tumor predominantly affects pediatric populations, whereas transitional cell carcinoma of the renal pelvis arises at the pelvicalyceal junction and is characterized by urothelial differentiation. At the molecular pathological level, ccRCC—the predominant subtype of renal cell carcinoma—is closely associated with mutations in the von Hippel-Lindau (VHL) gene [6,7]. In current clinical practice, computed tomography (CT) [8], magnetic resonance imaging, and ultrasonography [9,10] constitute the principal imaging modalities for evaluating renal lesions. Because most patients with kidney tumors lack specific early symptoms and are frequently diagnosed at an advanced stage, early detection and accurate imaging-based diagnosis are of paramount clinical importance. However, visual lesion detection alone is insufficient to guide complex therapeutic decision-making. Following confirmed or highly suspected diagnosis, precise 3D reconstruction [11] and quantitative analysis of the tumor region are fundamental prerequisites for surgical planning, treatment response assessment, and prognostication. Yet conventional clinical workflows rely on manual tumor delineation by radiologists—a process that is not only time-consuming and labor-intensive, but also susceptible to interobserver variability arising from subjective interpretation, potentially compromising the consistency and accuracy of treatment planning. Accordingly, the development of automated segmentation technology holds substantial significance for improving diagnostic efficiency, guiding surgical planning, and optimizing therapeutic strategies.

Early landmark contributions in this field focused primarily on enhancing feature extraction capability through architectural innovation. Yu et al [12] proposed Crossbar-Net, which introduced crossbar-shaped patch sampling and a cascaded training strategy to strengthen the capture of local textural features, achieving a Dice similarity coefficient (DSC) of 0.91 on kidney tumor segmentation tasks. To address organ boundary ambiguity in arterial-phase 3D CT scans, Myronenko and Hatamizadeh [13] designed a boundary-aware fully convolutional network that incorporated explicit edge supervision signals, elevating the joint kidney-tumor segmentation DSC to above 0.89 and establishing the importance of boundary constraints in fine-grained segmentation. This trajectory was further substantiated by studies in the KiTS challenge series: Heller et al [14] and Zhao et al [15] demonstrated, through ensemble optimization strategies and multiscale supervised U‑Net (MSS U-Net), respectively, that multiscale contextual information is critical for overcoming tumor morphological heterogeneity, with kidney and tumor DSC on the KiTS19 dataset consistently exceeding 0.97 and 0.80.

To address the persistent challenge of detecting small positive regions under limited supervision, subsequent research integrated residual connections with attention mechanisms. Guo et al [16] proposed residual and attention U-Net (RAU-Net), and Zhao et al [17] introduced boundary attention U-Net (BAU-Net), both of which used main-auxiliary branch synergy and weighted loss functions to substantially improve model sensitivity to small lesions. Notably, BAU-Net achieved kidney DSC of 0.98 and tumor DSC of 0.84 in the KiTS2021 challenge, marking the maturation of boundary attention mechanisms. More recent research has converged on 2 emerging directions. The first addresses the efficiency-interpretability trade-offs, exemplified by UNet-PWP developed by Rao et al [18], which reduces computational complexity through pretrained weight transfer and adaptive partitioning while incorporating explainable AI to enhance clinical trustworthiness. Along this line, recent studies have incorporated Grad-CAM and attention-based visualization techniques within EfficientNetV2-based frameworks to provide interpretable segmentation results across both clinical and public datasets [19]. Furthermore, combining Vision Transformers (ViTs) with conditional random fields (CRFs) has been shown to enhance boundary refinement while maintaining model transparency, offering a promising direction for explainable segmentation systems [20]. The second direction centers on global context modeling, as demonstrated by the simplified UNETR developed by Choi et al [21], which leverages the long-range dependency capture of Transformer architectures and integrates organ-level prior information for simultaneous segmentation, confirming that anatomical context integration significantly improves tumor localization accuracy [22]. Recent advances have further explored hybrid architectures that combine convolutional neural networks (CNNs) with ViTs to leverage both local feature extraction and global context modeling. For instance, multiscale fusion strategies integrated with UNet and Transformer backbones have demonstrated superior performance in capturing heterogeneous tumor structures [23]. In addition, cascaded Transformer frameworks incorporating mechanisms such as Gumbel-Softmax have been proposed to improve multiclass segmentation by enhancing feature selection and stage-wise refinement [24]. Analogous findings in brain tumor segmentation have demonstrated that backbone selection critically influences detection accuracy [25], and that integrating DeepLabV3+ with attention mechanisms yields consistent improvements across both public benchmarks and private clinical datasets [26], further supporting the design rationale of this study.

Despite impressive benchmark performance reported by these methods [27,28], their clinical translation remains substantially constrained. Prevailing high-performance approaches predominantly rely on 3D cascaded architectures or heavyweight Transformer models. Although 3D convolutions fully exploit volumetric spatial continuity, their substantial graphics processing unit (GPU) memory requirements and high computational overhead introduce considerable inference latency, precluding real-time deployment on standard clinical workstations or in time-critical emergency settings. Furthermore, complex cascaded pipelines increase system instability and maintenance burden. In response to this accuracy-efficiency dilemma, Lin et al [29] proposed an efficient 2D segmentation framework designed to transcend the prevailing 3D paradigm by optimizing receptive field design and feature aggregation in 2D CNNs, thereby substantially reducing computational complexity while preserving high segmentation accuracy for small and morphologically complex kidney tumors.

Notwithstanding the accuracy benchmarks established by the aforementioned 3D and hybrid architectures, their high computational cost and complex deployment pipelines remain primary barriers to clinical translation. Specifically, existing mainstream methods exhibit several key limitations: first, the prohibitive GPU memory demands of 3D convolutions restrict real-time inference on standard medical hardware [30], rendering them incompatible with the low-latency requirements of emergency care or intraoperative navigation; second, heavyweight Transformer models [31] often require large-scale pretraining data and have been reported to be more susceptible to missed detections of small lesions under oversmoothing or receptive field mismatch [32]; third, most methods fail to fully exploit deep semantic context at the 2D slice level during feature fusion, resulting in insufficiently sharp segmentation boundaries in challenging scenarios characterized by low tumor-to-background contrast and indistinct margins.

**Figure 1.**
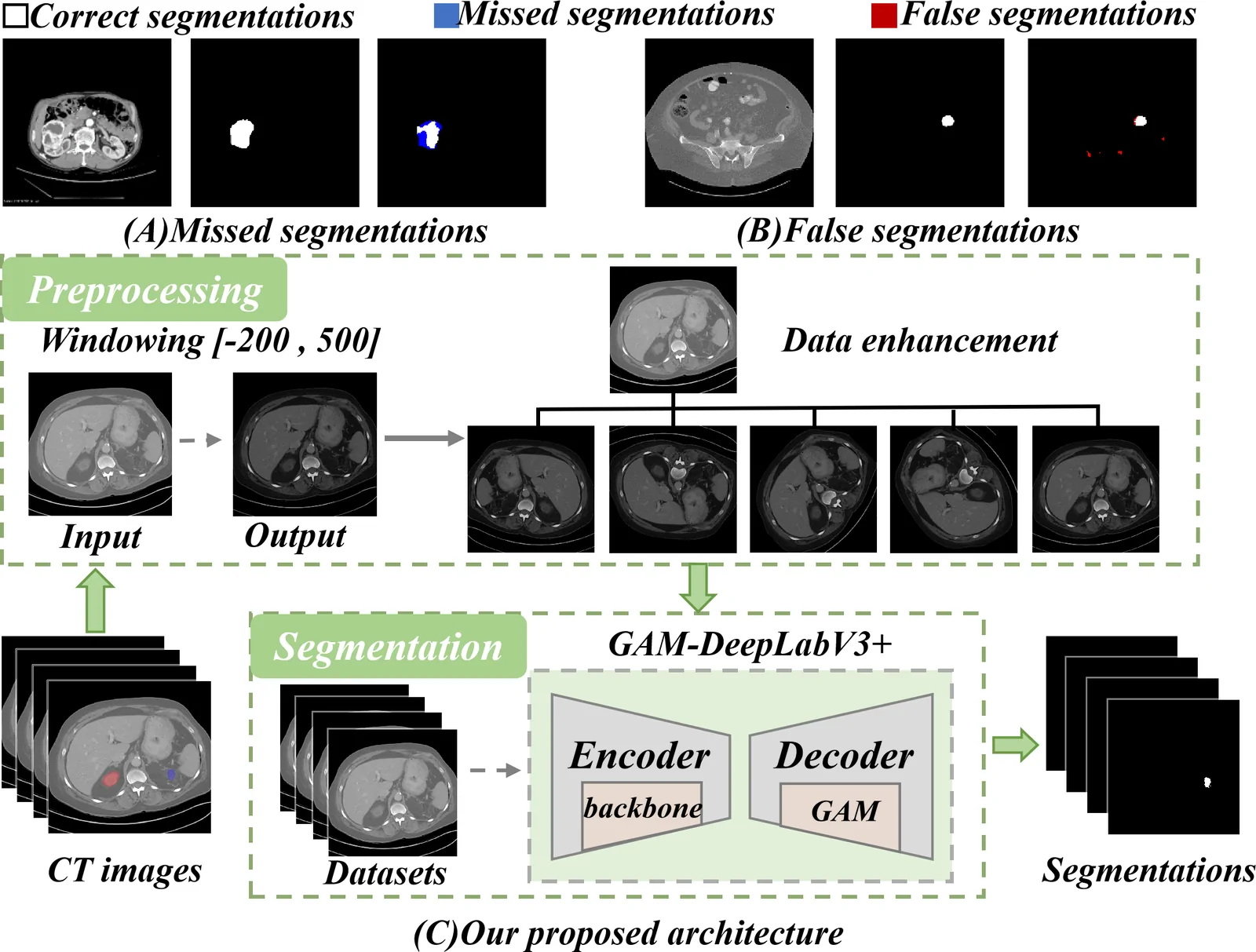
An overview of the complete technical pipeline and workflow of this study. CT: computed tomography; GAM: Global Attention Mechanism.

This study presents GAM-DeepLabV3+, a framework based on DeepLabV3+ for automated renal tumor segmentation (Figure 1), designed to reconcile the trade-off between accuracy and efficiency and to enhance the segmentation of small, low-contrast tumors. The system enhances the capture of semantic contextual information in tumor regions through backbone network refinement and the introduction of a global attention module. A targeted feature fusion strategy is further devised with the aim of improving tumor boundary delineation in complex backgrounds, including challenging cases such as small kidney tumors. The specific contributions of this work are as follows:

1. To reduce model parameter count, a lightweight enhanced MobileNetV2 is adopted as the backbone network, thereby strengthening the low-level feature extraction capability of GAM-DeepLabV3+.
2. Two Global Attention Mechanism (GAM) modules are introduced at key positions within the decoder: one is placed after a 1×1 convolution and prior to feature concatenation, while the other is embedded within the upsampling pathway. This configuration enables efficient fusion of deep and shallow features, enhancing the representation of salient information while suppressing background noise.
3. A BCEDiceLoss function is used to simultaneously mitigate class imbalance and refine boundary detail optimization. The proposed method achieves a mean Dice coefficient of 0.939 (SD 0.008) and 0.928 (SD 0.006) on a private dataset and the KiTS19 preoperative CT dataset, respectively, demonstrating its capacity for accurate renal tumor segmentation.

## Methods

### Overview

This section systematically presents a 2D segmentation method for renal tumors based on CT images. First, we provide a detailed description of the construction of the experimental dataset and the standardized preprocessing pipeline. Subsequently, we comprehensively describe the design and implementation of the DeepLabV3+ framework along with its novel modules.

### Description of Experimental Data

**Figure 2.**
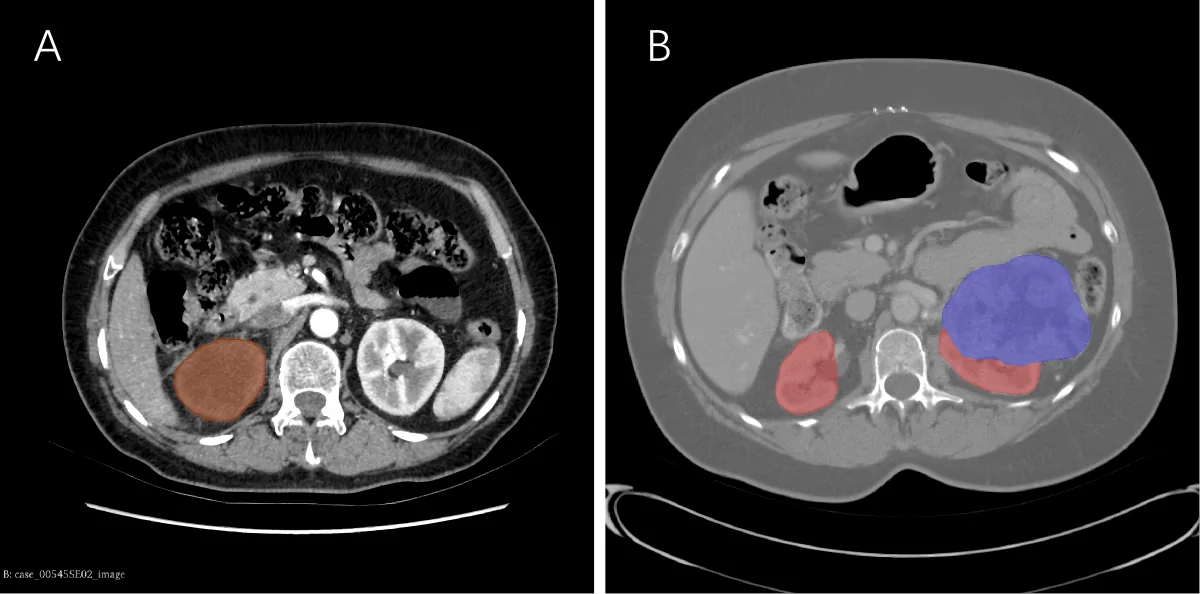
An example cross-sectional image showing the annotations of organs and tumors in our dataset: in the private dataset, renal tumors are highlighted in orange (A), while in the public dataset, kidneys are marked in red and renal tumors in blue (B).

In this study, we conducted a retrospective analysis using a private dataset obtained from the Lanzhou University Second Hospital. The dataset comprises 3D CT renal tumor images from 218 patients who were treated between 2016 and 2022. Data collection for this study was conducted over the 6-month period beginning on October 28, 2022. Notably, the dataset used in this study does not include any personal identifying information of the patients. The dataset is provided in Neuroimaging Informatics Technology Initiative (NIfTI) format with corresponding segmentation labels. In addition to CT imaging data, it encompasses various histological subtypes of renal tumors, including ccRCC, papillary renal cell carcinoma (pRCC), and chromophobe renal cell carcinoma (chRCC). The tumors were precisely classified into stages I, II, III, and IV based on the international TNM staging system. Moreover, the dataset contains fundamental clinical attributes such as patient age and gender; however, these clinical variables were collected but not used in the current segmentation algorithm, which operates solely on 2D CT images. The segmentation annotations were manually performed by experienced radiologists based on surgical pathology results to guarantee accurate tumor localization. Nontumor cystic lesions were strictly excluded to enhance the dataset’s quality. The annotations include 2 categories: background and renal tumor, as illustrated in Figure 2A.

Additionally, experiments were conducted on the publicly available KiTS19 dataset from the 2019 Kidney and Kidney Tumor Segmentation Challenge [33]. This dataset comprises original abdominal CT images and manually annotated label images by physicians, with annotation classes for background, kidney, and renal tumor, as illustrated in Figure 2B. To enhance data complexity and diversity, and to create a more challenging training environment for the network, we incorporated diverse cases from 210 patients. Notably, this dataset is publicly available on GitHub [34] and has been approved by the Institutional Review Board of the University of Minnesota. Throughout this study, we strictly complied with the terms of the KiTS19 license [33].

The private dataset and the public KiTS19 dataset exhibit complementary characteristics in terms of data construction, collectively offering diverse perspectives for evaluating model performance. The private dataset was meticulously annotated on a per-case basis by experienced radiologists, and a single representative slice was extracted for each patient using dedicated software from 3D volumetric NIfTI-format data. In contrast, the KiTS19 dataset was preprocessed using the official pipeline, generating a series of continuous slices from the raw volumetric scans. To comprehensively assess the generalizability of the proposed model, we conducted experiments on both datasets, thereby validating its robustness and adaptability across different data acquisition and annotation protocols.

### Ethical Considerations

This study was approved by the Ethics Committee of the Second Hospital of Lanzhou University (approval number: 2022A-575), ensuring compliance with medical ethical standards. Given the retrospective nature of the study and the use of deidentified data, the requirement for informed consent was waived by the ethics committee. All imaging data were fully anonymized prior to analysis, and no direct or indirect patient identifiers were accessible to the research team. No financial or material compensation was provided to participants for their inclusion in this study. No images included in the paper or supplementary materials contain identifiable information of individual participants.

### Data Preprocessing

#### Overview

In the data preprocessing stage, we use a variety of strategies—including image slicing, window width and level adjustment, data augmentation, and label binarization—to enhance both the quality of the training dataset and the efficiency of the deep learning network training. These measures enable the model to better adapt to the feature distribution of the training data. Through data augmentation, not only can more diverse and complex training samples be generated, but the model’s generalization ability is also significantly improved, effectively preventing overfitting [35]. Furthermore, to ensure consistency in data processing, all images are uniformly preprocessed to a resolution of 512×512 pixels for input.

#### Windowing

**Figure 3.**
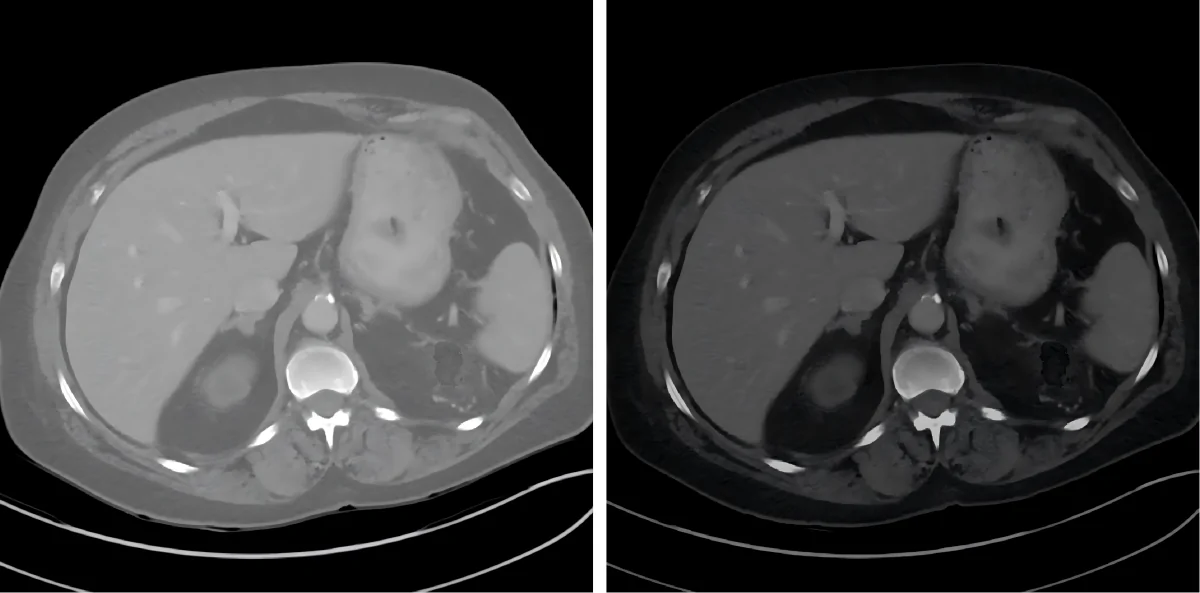
Comparison diagram for window width and window level adjustments.

Since the experimental dataset consists of 3D medical imaging data, while our segmentation network operates on a 2D architecture, it is necessary to first convert the data from 3D to 2D. For the private dataset, we used the widely used 3D Slicer software to precisely extract renal tumor slices. Meanwhile, for the KiTS19 public dataset, we used the official open-source script provided by the competition organizers to perform automated batch slicing. Only 2D CT slices containing renal tumors were retained. For the KiTS19 dataset, labels were converted to a binary schema: the kidney label (class 1) was merged into the background (class 0), retaining only background and renal tumor (class 2) categories, consistent with the annotation schema of the private dataset, while those without labeled tumors were discarded. After preprocessing, the private dataset and KiTS19 dataset yielded 218 and 4800 renal tumor images, respectively. To enhance the visibility of target tissues, we adjusted the window width (WW) and window level (WL) during the slicing process. First, we applied a dual-threshold truncation method to constrain CT values within [−200, 500] HU, effectively reducing the impact of extreme values. This range was selected to preserve contextual anatomical information from surrounding structures, which assists in localizing the kidney and tumor relative to adjacent tissues, and is consistent with preprocessing choices in prior KiTS19 studies. Next, we determined a dynamic effective grayscale range based on the 0.5%‐99.5% quantiles and applied a Gaussian kernel filter to suppress high-frequency noise. Finally, each CT volume was standardized using *z*-score normalization by subtracting the volume mean and dividing by the SD. Min-max scaling was then applied to rescale the pixel values to the range [0,1] for network input. These preprocessing steps significantly improved the deep network’s ability to extract meaningful features, as shown in Figure 3.

#### Data Enhancements

**Figure 4.**
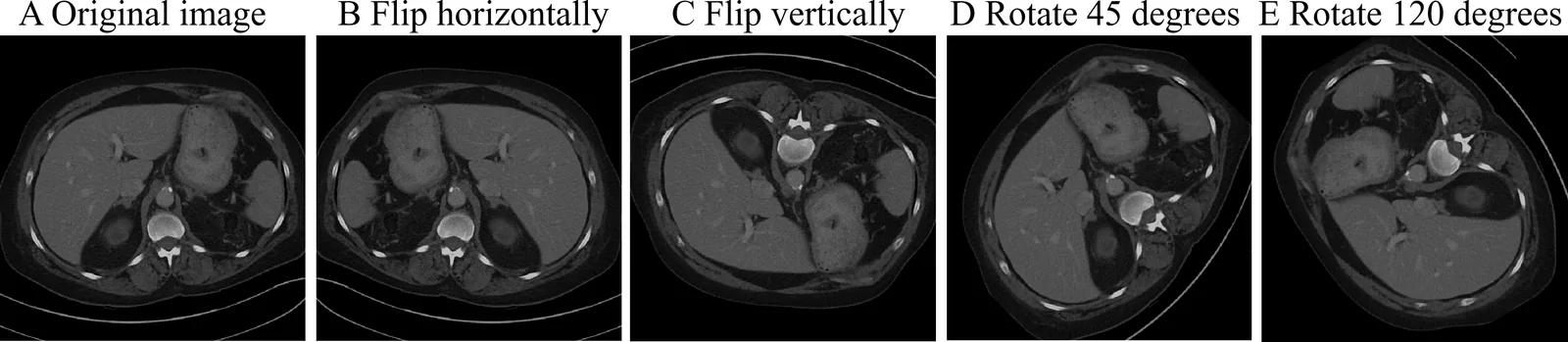
Data enhancement.

To mitigate overfitting and enhance the model’s generalization ability, we used various data augmentation techniques, including flipping and rotation. By skillfully leveraging artificial expansion, we significantly enriched the diversity of the training data, thereby effectively reducing the adverse impact of data scarcity on model performance, as illustrated in Figure 4. During training, dynamic data augmentation is applied to each CT image using random flipping and multiangle rotations. Moreover, these transformations are randomly applied to all images with a 50% probability to ensure that the augmented data generated are sufficiently diverse and representative.

#### Kidney Tumor Segmentation

This section provides a detailed description of the overall architecture for automatic segmentation of renal tumors in CT images. First, in the “GAM-DeepLabV3+ Modeling” section, we introduce the overall system design and elaborate on the implementation details of the encoder and decoder, respectively; next, in the “MobileNetV2 Module” section, we focus on the lightweight improvements made to the MobileNetV2 network structure; finally, in the “GAM Module” section, we delve into the design principles and functional mechanisms of the GAM module.

#### GAM-DeepLabV3+ Modeling

**Figure 5.**
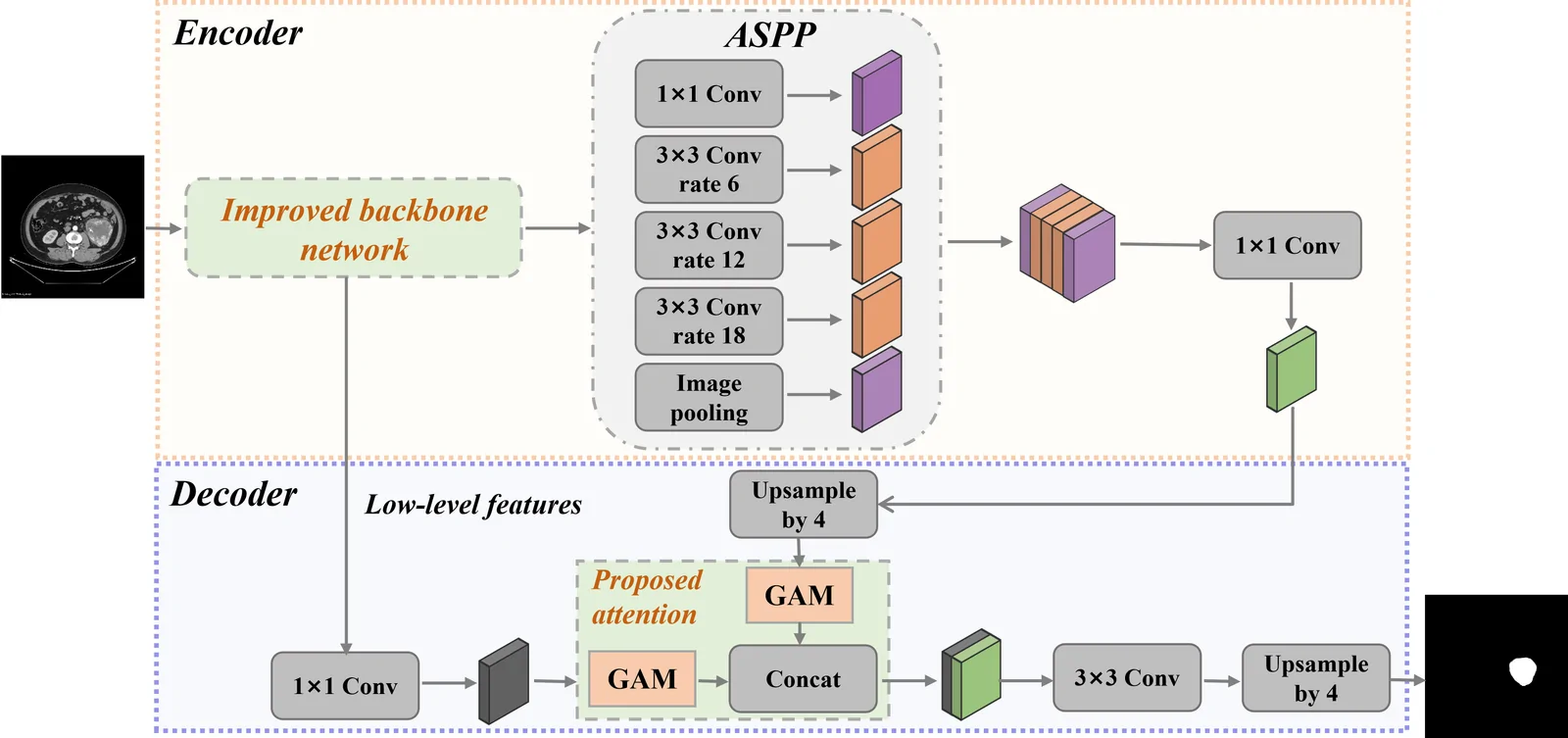
GAM-DeepLabV3+ architecture. ASPP: Atrous Spatial Pyramid Pooling; Conv: convolution; GAM: Global Attention Mechanism.

The proposed GAM-DeepLabV3+ integrates three key components, each addressing a specific limitation of the standard DeepLabV3+: (1) the improved MobileNetV2 backbone with Efficient Channel Attention (ECA) reduces model parameters while enhancing low-level feature extraction; (2) the GAM modules in the decoder improve multiscale feature fusion and suppress background interference; and (3) the BCEDiceLoss function addresses class imbalance while optimizing boundary delineation. In recent years, DeepLabV3+ has demonstrated outstanding performance in multiscale feature extraction and fusion [36]. Its encoder-decoder structure is particularly effective in segmentation tasks. However, the traditional DeepLabV3+ encoder suffers from missing small targets, while its decoder is suboptimal in capturing global information, allocating feature weights, and resisting noise, which to some extent limits segmentation accuracy. To address these limitations, we propose an improved segmentation algorithm, GAM-DeepLabV3+, the architecture of which is depicted in Figure 5.

Specifically, in the encoder section, a CT image with dimensions of 512×512×3 is first processed by the backbone network for preliminary feature extraction. In contrast to the conventional use of the Xception network [37], we introduce a lightweight MobileNetV2 network [38] to extract both shallow and deep features. This replacement significantly reduces the computational burden while further optimizing the feature extraction process through depthwise separable convolutions, inverted residual structures, and linear bottleneck designs, which are particularly effective in preserving fine-grained information and improving the detection of small targets. Subsequently, the high-level features are fed into the Atrous Spatial Pyramid Pooling (ASPP) module [39]. The ASPP module, composed of 4 convolutional layers with dilation rates of 1, 6, 12, and 18, respectively, along with a global average pooling operation, effectively integrates contextual information from different receptive fields, providing a rich and precise feature foundation for the subsequent decoder stage.

In the decoder, we introduce GAM modules at 2 critical locations (as shown in Figure 5) to enhance the fusion and selection of multiscale features. First, features obtained from the encoder’s lower layers are compressed via a 1×1 convolution and initially fused with high-level features; after upsampling, they are input to the first GAM module [40] to highlight key information and suppress background noise. Subsequently, these attention-optimized features are merged with another set of high-level features in the second GAM module, and the results are integrated with earlier processed features via concatenation. Finally, the fused features undergo a 3×3 convolution and further upsampling to progressively restore resolution, ultimately producing segmentation results that match the original image dimensions. By introducing GAM modules at these 2 positions, the network is designed to better attend to edge details and to exploit global and local information at different resolutions, thereby aiming to improve segmentation accuracy and robustness while maintaining a lightweight model.

#### MobileNetV2 Module

##### Overview

**Figure 6.**
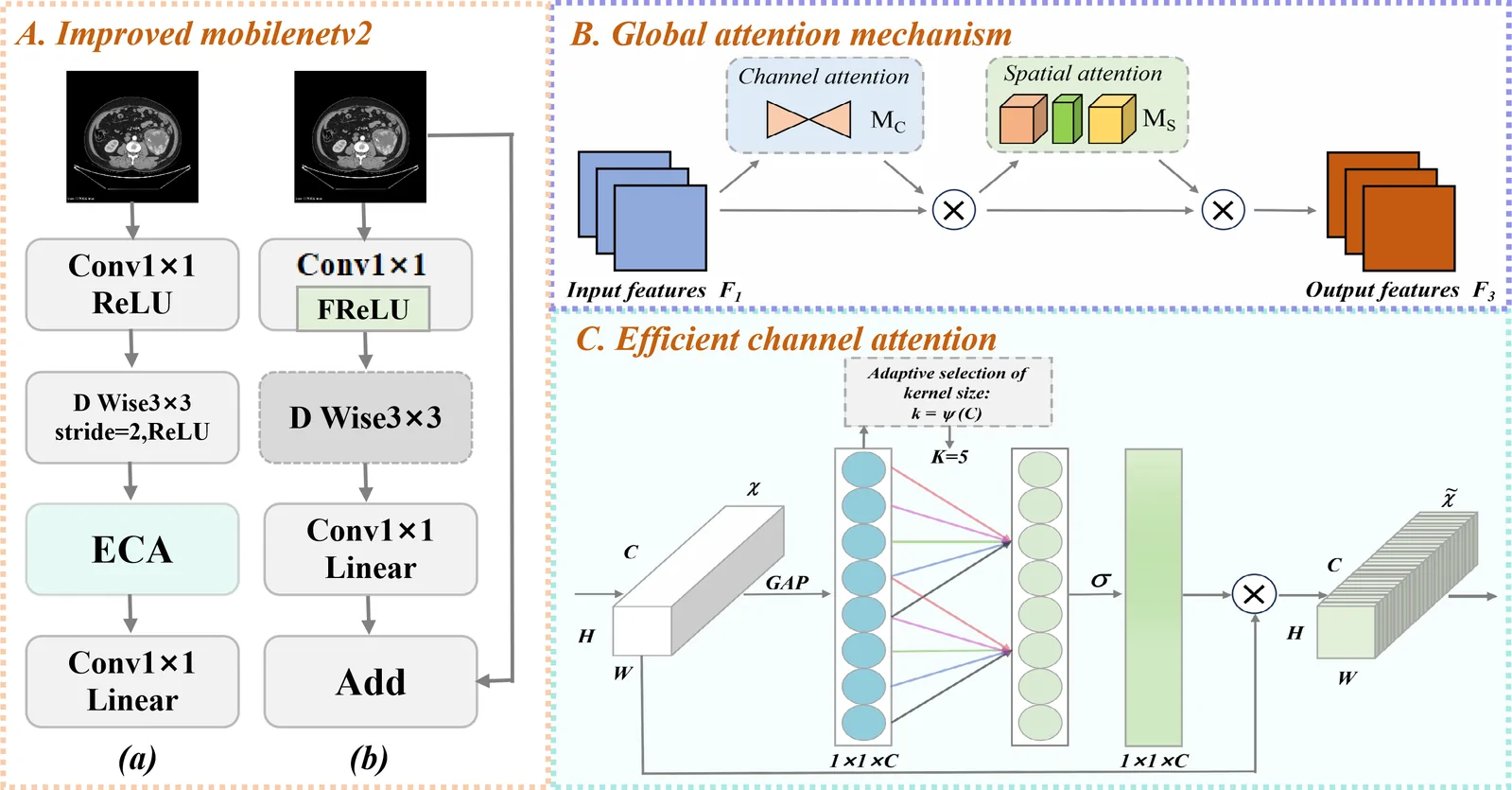
Detailed architectures of key modules in the proposed GAM-DeepLabV3+ framework. Conv: convolution; ECA: Efficient Channel Attention; FReLU: free rectified linear unit; GAP: Global Average Pooling; ReLU: rectified linear unit.

Addressing the dual requirements of network lightweight design and effective feature extraction for high-resolution medical image segmentation tasks, we adopt MobileNetV2 as the backbone encoder and optimize it accordingly. Based on depthwise separable convolutions and inverted residual blocks, MobileNetV2 implements an “expansion–extraction–projection” feature processing paradigm that balances computational complexity and feature representation, as illustrated in Figure 6. Initially, a 1×1 convolution expands the low-dimensional input into a high-dimensional feature space; then, a 3×3 depthwise convolution extracts spatial features; finally, a 1×1 convolution projects the features back to a lower dimension. In contrast to traditional residual structures with nonlinear transformations, MobileNetV2 introduces a linear bottleneck in its inverted residual blocks, which suppresses redundant nonlinear activations in high-dimensional features. This not only ensures stable gradient propagation but also provides a structural basis for preserving fine feature details.

Although MobileNetV2 exhibits efficiency in feature extraction, there remains room for improvement in the semantic discrimination of channel dimensions. To address this, we propose a hierarchical optimization strategy. In the low-level feature extraction stage, an ECA module [41] is embedded after the depthwise separable convolution to enhance the expression of fundamental features such as edges and textures through adaptive channel weighting. Then, in the high-level feature processing stage, an improved free rectified linear unit (FReLU) activation function combined with a residual connection is used to optimize the gradient propagation path while preserving the integrity of the original information. This hierarchical enhancement strategy creates a complementary effect: the former calibrates channel semantics to improve local detail representation, and the latter strengthens global semantic modeling through improved nonlinear transformations. Together, they construct a hierarchical fine-grained feature expression system that maintains the network’s lightweight advantage. The subsequent sections will discuss these 2 improvement mechanisms in detail.

##### Low-Level Feature Improvement: Embedding of ECA Modules

In the inverted residual blocks of MobileNetV2, low-level features are typically extracted using 3×3 depthwise separable convolutions. However, this process primarily relies on local operations within each channel and lacks global modeling of interchannel relationships. To address this limitation, we introduce an ECA module between the 3×3 depthwise convolution (Dw Conv) and the subsequent 1×1 projection convolution. The purpose is to perform adaptive reweighting of channel information, thereby enhancing the network’s ability to capture critical low-level features such as edges and textures.

The ECA module is designed to capture cross-channel dependencies through a lightweight attention mechanism, thus improving the discriminative power of feature representations. The detailed computational process of the ECA module is illustrated in Figure 6. For an input feature map$X\in R^{C\times H\times W}$, the ECA first compresses the spatial information into a channel descriptor vector $g\in R^{C}$via Global Average Pooling (GAP):


 (1) $$E_{r}=\frac{1}{J}\times \frac{1}{N^{2}}\sum_{i=1}^{N}\sum_{j=1}^{N}x_{j}\;\ell (i,j)$$

Subsequently, this descriptor vector is fed into a 1D convolution (Conv1D) over the channel dimension. The kernel size *k* is adaptively determined according to the channel number *C* as k=ψ(c) , where ψ(c) denotes the nearest odd integer to $\left|(\log_{2}(c)+b)/\gamma \right|$, with *γ*=2 and b=1 in our implementation. This adaptive Conv1D captures local cross-channel interactions, and a Sigmoid activation function is then applied to generate channel attention weights $w\in R^{C}$:


(2) w=σ(Conv1D(g;k))

Finally, these weights are multiplied with the original feature map on a per-channel basis, yielding the enhanced feature representation:


(3) $$X^{\prime }=w⊗X$$

Here, ⊗ denotes element-wise multiplication across channels. In this formulation, $X\in R^{C\times H\times W}$ denotes the input feature map with *C* channels, spatial height *H*, and width *W*; $g\in R^{C}$ is the channel descriptor vector obtained via global average pooling; $w\in R^{C}$ represents the learned channel attention weights generated by the Conv1D and Sigmoid operations; and *X*’ is the channel-recalibrated output feature map. This mechanism eliminates the need for an explicit fully connected layer, thereby reducing the number of parameters while maintaining computational efficiency. Moreover, the fine-grained channel reweighting effectively enhances the model’s responsiveness to key information.

##### Advanced Feature Improvement: FReLU Activation and Residual Connection

In the high-level feature extraction stage, to further enhance nonlinear expressive capability and ensure stable information propagation, we adopt an improved activation function, FReLU, and incorporate residual connections within the inverted residual blocks. Unlike the traditional rectified linear unit (ReLU) activation, which truncates negative information, FReLU uses local spatial information to generate a nonlinear mapping, thereby increasing the flexibility of feature expression. Its specific formulation can be expressed as follows:


(4) FReLU(x)=max{x,ϕ(x)}

Here, ϕ(x) generated by the local convolution operation adaptively captures local feature information. In this way, not only is the positive information of the original input *x* preserved, but auxiliary information derived from local spatial awareness is also introduced. Furthermore, the residual connection in the inverted residual block adds the input feature *X* to the output *F*(*X*) obtained after a series of convolution and activation operations. This ensures stable gradient propagation and effectively preserves the initial feature information:


 (5) H(X)=X+F(X)

Here, *X* denotes the input feature map of the inverted residual block; *F*(*X*) represents the transformed feature obtained through a series of convolution, normalization, and activation operations; and *H*(*X*) denotes the residual output obtained by adding the original input *X* to the transformed feature *F*(*X*). This residual formulation preserves the original feature information while facilitating gradient propagation, thereby alleviating the gradient vanishing problem in deep networks. As a result, high-level features can retain richer semantic information after multiple nonlinear transformations, which is expected to support fine-grained segmentation.

##### GAM Module

In renal tumor segmentation tasks, models in the decoder stage are often affected by inconsistent fusion of features from different resolutions and noise interference, which may result in misclassification of some critical features and ultimately compromise segmentation performance. To address this issue, we introduce the GAM module to enhance focus on target regions during feature fusion, as shown in Figure 6. Given that both channel and spatial information are essential for refining and restoring resolution, the GAM module is incorporated at 2 critical locations within the decoder, following the 1×1 convolution and during the 4× upsampling. This placement enables the extraction of both channel and spatial features from the image.

The design inspiration for the GAM module comes from the remarkable performance of attention mechanisms in information filtering. Its structure mainly comprises 3 components: a channel attention branch, a spatial attention branch, and a residual connection, which together enable the collaborative modeling of global semantic information and local detail.

In the channel attention branch, the module first applies global average pooling to the input feature map to obtain global response information for each channel. In our implementation, the channel attention branch uses a multilayer perceptron (MLP) structure with a channel reduction ratio of 4 to model interchannel dependencies and generate importance weights for each channel. These weights reflect the contribution of each channel to the segmentation task and are multiplied element-wise with the original feature map *F* to obtain the channel-enhanced feature map $F_{c}$, thereby reinforcing critical channel information and suppressing redundant features:


(6) $$F_{c}=M_{c}⊗F$$

Meanwhile, the spatial attention branch focuses on capturing local contextual information at each spatial location of the feature map. In our implementation, this branch consists of 2 successive 7×7 convolutional layers with padding of 3. The first convolution reduces the channel dimension from C to C/4, followed by batch normalization and ReLU activation, while the second convolution restores the channel dimension from C/4 to C and is followed by batch normalization. The channel reduction ratio in the spatial attention branch was set to 4. After these convolution and normalization operations, the spatial attention branch produces a spatial weight map $M_{s}$ that matches the dimensions of the input feature map. This spatial weight map is then multiplied element-wise with *F*_*C*_ to obtain the spatially enhanced feature map *F*_*S*_. Through dynamic adjustment of features at different spatial positions, the saliency of the target region is further enhanced:


(7) $$F_{s}=M_{s}⊗F_{c}$$

Finally, to ensure stable propagation of features during the attention processing, the GAM module incorporates a residual connection. After the channel and spatial attention processing, the optimized features are added to the original input features, achieving shortcut information transmission. This design not only helps mitigate the gradient vanishing problem in deep networks but also preserves key information from the original features, ensuring that the introduction of the attention mechanism does not result in loss of information, thereby enhancing the overall robustness of feature representation. The schematic of this structure is shown in Figure 6.

Overall, by jointly applying channel and spatial attention mechanisms, the GAM module adaptively weights the input feature map, significantly enhancing focus on the target region and improving the quality of feature fusion while maintaining a lightweight model. Its flexible and efficient architecture not only boosts the model’s capability to extract fine-grained information but also provides robust theoretical support and technical assurance for segmentation in complex scenarios.

### Evaluation Metrics

Fundamentally, image segmentation can be viewed as a pixel-level classification problem, where each pixel is classified as either background or target. For segmentation tasks, it is ideal for the network to correctly predict target pixels as true positives (TP) and background pixels as true negatives (TN), whereas misclassifying background pixels as target or vice versa results in false positives (FP) and false negatives (FN), respectively. In this study, the performance of the proposed GAM-DeepLabV3+ network in renal tumor segmentation is quantitatively evaluated using 6 metrics: intersection over union (IoU), Dice, recall, precision, accuracy, and 95% Hausdorff distance (HD95). IoU is a widely used metric in image segmentation that calculates the ratio of the intersection to the union of the predicted segmentation and the ground truth, as given by the following formula:


(8) $$IoU=\frac{TP}{TP+FP+FN}$$

The Dice coefficient, which measures the similarity between 2 samples on a scale from 0 to 1 (with values closer to 1 indicating better performance), is calculated as follows:


(9) $$DSC=\frac{2TP}{2TP+FP+FN}$$

Recall is defined as the proportion of TP pixels correctly identified by the model relative to all actual positive pixels in the ground truth, computed as:


(10) Recall=TPTP+FN

Precision refers to the ratio of TP pixels among all pixels predicted as positive by the model, computed as:


(11) $$\mathrm{Pr}ecision=\frac{TP}{TP+FP}$$

Accuracy denotes the proportion of all pixels that are correctly classified by the model, measuring the overall prediction accuracy with the formula:


 (12) $$ACC=\frac{TP+TN}{TP+FP+FN+TN}$$

HD95 refers to the 95th percentile Hausdorff distance between the predicted segmentation boundary and the ground-truth boundary. It is less sensitive to extreme outliers than the maximum Hausdorff distance and was used to evaluate boundary accuracy. It is calculated as follows:


(13) $$\begin{array}{rl} HD95(A,B) & =\max\{P95(d(A,B)),\\ \;P95(d(B,A))\} \end{array}$$

where $d(A,B)=\left\{\min\{\|b-a{\|}_{2}:a\in A\}\right\}\;\text{and}\;d(B,A)=\left\{\min\{\|b-a{\|}_{2}:b\in B\}\right\}$. Here, A and B denote the boundary point sets of the predicted segmentation and ground-truth mask, respectively. In this study, HD95 was reported in pixels.

Our proposed loss function is a combination of binary cross-entropy (BCE) loss and Dice loss, referred to as BCEDiceLoss. BCE loss, a special case of cross-entropy loss suitable for binary classification problems, is computed as follows:


(14) BCE=−[ylog(p)+(1−y)log(1−p)]

Here, y represents the ground truth label (0 or 1), and p denotes the model’s predicted probability. The BCE loss function quantifies the discrepancy between the ground truth and the predicted probabilities.

By combining BCE loss with Dice loss, both classification accuracy and segmentation detail can be simultaneously optimized during training. Specifically, the total loss can be computed using the following formula:


(15) BCEDiceLoss=BCE+λ(1−Dice)

Here, BCE refers to the binary cross-entropy loss, and Dice denotes the Dice coefficient (computed with a smoothing term), so that the term (1 − Dice) constitutes the Dice loss and a higher Dice coefficient yields a lower loss. λ is a hyperparameter used to balance the contributions of the 2 loss terms, which was empirically set to 2 in all experiments. During training, the model optimizes both BCE and Dice objectives concurrently, thereby improving its overall performance.

By integrating the metrics, we can comprehensively and objectively evaluate the model’s performance under various conditions, thereby providing robust data support for subsequent model optimization and clinical applications.

## Results

### Experimental Settings

In our experiments, we used the CT data described in the data preparation section and established the deep learning environment using Python 3.8 (Python Software Foundation) and PyTorch 1.11.0 (PyTorch Foundation, The Linux Foundation) on Ubuntu 20.04 (Canonical Ltd). The hardware configuration included an AMD EPYC 9754 processor and an RTX 4090D GPU with 24 GB of memory. During training, the batch size was set to 8 and the maximum number of epochs was set to 200, with validation performed at the end of each epoch. An early stopping strategy was used to prevent overfitting. The Adam optimizer was adopted with an initial learning rate of 1×10^–4^, a weight decay of 1×10^–4^, *β*1 of .9, and a minimum learning rate of 1×10^–5^. To ensure reproducibility and assess result stability, all compared models were trained and evaluated 5 times using different random seeds (42, 123, 456, 789, and 1024), and the results are reported as mean (SD). All dataset splits were performed at the patient level to prevent data leakage. For the private dataset, 218 patients were divided into 153 training, 43 validation, and 22 testing cases, with one representative slice extracted per patient. For the KiTS19 dataset, 210 patients were divided into 147 training, 42 validation, and 21 testing cases; each patient contributed multiple continuous slices containing tumor regions, resulting in 4800 slices for training and evaluation. All backbone networks used in the ablation experiments were initialized with ImageNet pretrained weights. For the no-new-Net (nnU-Net) baseline, we adopted a 2D nnU-Net implementation and trained it under the same unified experimental protocol as the other baseline models. The complete self-configuring nnU-Net pipeline, including automated experiment planning, preprocessing, configuration selection, and postprocessing, was not used. To assess statistical significance, paired *t* tests were conducted across the 5 independent runs for Dice scores between the proposed method and baseline models, with statistical significance defined as *P*<.001.

### Visualization Results

**Figure 7.**
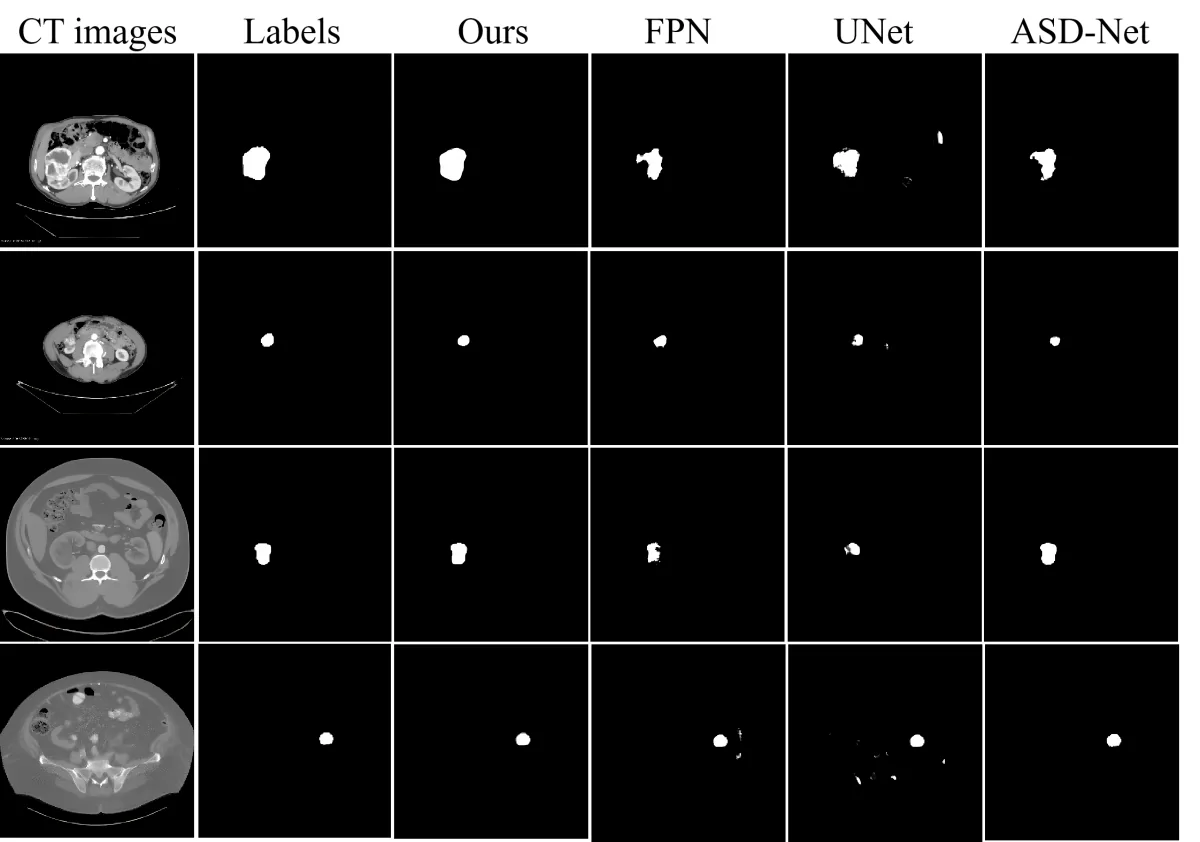
Visualization of the segmentation results of the proposed model and their comparison. CT: computed tomography; FPN: feature pyramid network.

During the evaluation of the proposed GAM-DeepLabV3+ model, training and testing were performed on both a private dataset and the public KiTS19 dataset. All splits were performed at the patient level, with 70% for training, 20% for validation, and 10% for testing, with identical hyperparameter settings and training strategies used to objectively compare the model’s performance across different data sources. The proposed model converged stably within 200 epochs on both datasets, with no signs of overfitting observed under the patient-level experimental setup. Figure 7 presents the final segmentation visualization results.

We randomly selected 2 groups of CT image predictions from the test sets of both the private dataset and the KiTS19 dataset for comparative analysis, as shown in Figure 7. This visual comparison demonstrates the segmentation results of different network models on renal tumors, with the white regions representing the tumor segmentation. It can be observed that traditional classic networks such as U-Net and feature pyramid network (FPN) produce relatively coarse segmentation boundaries for complex-shaped and small-volume tumor targets, often leading to segmentation errors due to insufficient deep feature extraction, which results in the failure to capture certain detailed regions. In contrast, the proposed GAM-DeepLabV3+ network performs well in segmenting small and irregular lesion areas. Compared with other classical networks, it reduces both undersegmentation and oversegmentation issues, primarily because the network is able to filter channels that contain critical information through dual attention modules, thereby reducing feature loss and enhancing overall accuracy. Consequently, it can, to a certain extent, resolve the separation between small tumor regions and the kidney, and compensate for missegmentation or omission errors observed in other networks, thereby providing valuable assistance for clinical diagnosis of renal tumors.

### Experimental Results

#### Overview

In our experiments, we compared the performance of the proposed network with several classical segmentation models (including U-Net, Attention-UNet, U-Net++, and ResUNet) on segmentation tasks using both a private dataset and the public KiTS19 dataset. Tables 1 and 2 present the detailed results of each model across various metrics.

**Table 1.** Comparison between GAM-DeepLabV3+ and other models (private dataset)^a^.

Model	IoU^b^, mean (SD)	Dice, mean (SD)	Recall, mean (SD)	Precision, mean (SD)	Accuracy, mean (SD)	HD95^c^, mean (SD)
ResUNet	0.730 (0.014)	0.820 (0.008)	0.860 (0.007)	0.784 (0.009)	0.980 (0.001)	5.964 (1.731)
UNet	0.744 (0.017)	0.832 (0.006)	0.881 (0.008)	0.783 (0.007)	0.981 (0.002)	5.218 (1.642)
UNet++	0.840 (0.0017)	0.865 (0.007)	0.899 (0.010)	0.890 (0.008)	0.983 (0.001)	3.287 (1.104)
Attention-UNet	0.859 (0.0011)	0.867 (0.005)	0.888 (0.006)	0.893 (0.007)	0.983 (0.001)	3.012 (0.876)
FPN^d^	0.866 (0.0010)	0.893 (0.008)	0.911 (0.013)	0.907 (0.005)	0.983 (0.002)	2.684 (0.932)
nnU-Net^e^ (2D, custom)	0.868 (0.020)	0.908 (0.013)	0.902 (0.015)	0.891 (0.014)	0.988 (0.002)	3.936 (0.842)
DeepLabV3+	0.906 (0.016)	0.911 (0.008)	0.913 (0.011)	0.917 (0.008)	0.987 (0.001)	2.956 (0.642)
Ours	0.928 (0.012)	0.939 (0.008)	0.941 (0.007)	0.943 (0.006)	0.999 (0.000)	1.485 (0.522)

a The nnU-Net baseline in this table was implemented as a 2D nnU-Net model and trained under the same unified experimental protocol as the other baseline models, including identical patient-level data splits, optimizer settings, batch size, early stopping strategy, and random seeds. The complete self-configuring nnU-Net pipeline, including automated experiment planning, preprocessing, configuration selection, and postprocessing, was not used.

b IoU: intersection over union.

c HD95: 95% Hausdorff distance.

d FPN: feature pyramid network.

e nnU-Net: no-new-Net.

**Table 2.** Comparison between GAM-DeepLabV3+ and other models (KiTS19)^a^.

Methods	IoU^b^, mean (SD)	Dice, mean (SD)	Recall, mean (SD)	Precision, mean (SD)	Accuracy, mean (SD)	HD95^c^, mean (SD)
ResUNet	0.872 (0.013)	0.906 (0.009)	0.881 (0.010)	0.927 (0.008)	0.987 (0.002)	3.842 (1.214)
FPN^d^	0.881 (0.011)	0.912 (0.008)	0.893 (0.012)	0.931 (0.007)	0.988 (0.002)	3.516 (1.038)
UNet	0.888 (0.010)	0.918 (0.007)	0.905 (0.009)	0.936 (0.006)	0.986 (0.001)	3.182 (0.964)
Attention-UNet	0.894 (0.009)	0.921 (0.006)	0.910 (0.008)	0.938 (0.006)	0.990 (0.001)	2.964 (0.887)
nnU-Net^e^ (2D, custom)	0.890 (0.010)	0.915 (0.007)	0.911 (0.009)	0.934 (0.006)	0.989 (0.002)	2.970 (0.850)
UNet++	0.890 (0.010)	0.919 (0.007)	0.907 (0.009)	0.937 (0.006)	0.988 (0.001)	3.041 (0.912)
DeepLabV3+	0.897 (0.011)	0.925 (0.007)	0.910 (0.009)	0.938 (0.006)	0.991 (0.001)	2.712 (0.803)
Ours	0.902 (0.008)	0.928 (0.006)	0.888 (0.007)	0.939 (0.005)	0.992 (0.001	2.536 (0.742)

a The nnU-Net baseline in this table was implemented as a 2D nnU-Net model and trained under the same unified experimental protocol as the other baseline models. Because our study used 2D slice-level binary tumor segmentation, patient-level data splits, tumor-containing slice selection, and a different evaluation protocol, these results should not be directly compared with the official KiTS19 challenge leaderboard or the tumor Dice score reported by Heller et al [33].

b IoU: intersection over union.

c HD95: 95% Hausdorff distance.

d FPN: feature pyramid network.

e nnU-Net: no-new-Net.

Note that the high accuracy value observed on the private dataset is expected because renal tumor segmentation is highly class-imbalanced, with background pixels occupying the vast majority of each CT slice. Therefore, accuracy may be inflated by correctly classified background pixels, and overlap-based metrics such as Dice and IoU provide more informative evaluation of tumor segmentation performance.

As can be seen from the data, our proposed GAM-DeepLabV3+ network achieved the best overall performance among the compared methods, particularly in terms of IoU, Dice, and HD95. Specifically, the IoU and Dice coefficients of our method on the renal tumor test set reached 92.8% and 93.9%, respectively, representing improvements of 6.2% and 4.6% over FPN. Moreover, compared with the baseline DeepLabV3+, our method achieved increases of 2.2% and 2.8% in IoU and Dice coefficients, respectively. Paired *t* tests based on the 5 independent runs showed that the Dice improvements of GAM-DeepLabV3+ over all compared baseline models on the private dataset were statistically significant (all *P*<.001).

Similarly, as shown in Table 2, our method outperforms the other models on the public KiTS19 dataset. Paired *t* tests based on the 5 independent runs showed that the Dice improvements of GAM-DeepLabV3+ over all compared baseline models on the KiTS19 dataset were statistically significant (all *P*<.01). On the test set, the IoU and Dice coefficients reached 90.2% and 92.8%, respectively, outperforming all compared baseline models on the KiTS19 external validation set, further demonstrating the strong generalizability of the proposed approach.

As shown in Table 3, GAM-DeepLabV3+ achieved a lower per-slice inference latency than the standard DeepLabV3+ on both the private and the public KiTS19 datasets, corresponding to a speed-up of approximately 1.5× on both datasets (private: from 21.8 to 14.2 ms/slice; KiTS19: from 21.8 to 14.4 ms/slice). These results highlight the efficiency and lightweight characteristics of the enhanced MobileNetV2 architecture. The experimental findings presented in the “Experimental Results” section systematically validate the effectiveness and practical value of the proposed method in terms of both segmentation accuracy and computational performance.

**Table 3.** Per-slice inference latency (ms; mean, SD over 5 runs; RTX 4090D; batch size 1).

Model	Private dataset (ms), mean (SD)	KiTS19 (ms), mean (SD)
DeepLabV3+	21.8 (0.4)	21.8 (0.8)
GAM-DeepLabV3+	14.2 (0.4)	14.4 (0.5)

All results are reported as mean (SD) over 5 runs. Inference time refers to pure model forward-pass computation, excluding data loading and preprocessing.

#### Ablation Experiment

In this section, we conducted a series of experiments to investigate the impact of different modules on the model’s performance, mainly examining how various backbone networks and attention mechanisms integrated within the DeepLabV3+ framework affect segmentation outcomes. Our experiments aim to verify the feasibility and effectiveness of the improved MobileNetV2 network and GAM module in enhancing renal tumor segmentation performance. To this end, all backbone networks (MobileNetV2, Xception, and ResNet101) were initialized with ImageNet-pretrained weights to ensure a fair comparison and avoid performance differences attributable to training-from-scratch instability on the small private dataset. We compared the performance of each variant using quantitative metrics. The results indicate that incorporating the GAM module on top of the lightweight, improved MobileNetV2 backbone significantly enhances overall segmentation accuracy; specific data are presented in Table 4.

**Table 4.** Ablation experiments (private dataset).

MobileNet V2	Xception	Resnet101	ECA^a^	CBAM^b^	GAM^c^	IoU^d^, mean (SD)	Dice, mean (SD)	Recall, mean (SD)	Precision, mean (SD)	Accuracy, mean (SD)	HD95^e^, mean (SD)
✓^f^						0.910 (0.014)	0.922 (0.008)	0.925 (0.009)	0.927 (0.007)	0.987 (0.001)	2.214 (0.801)
	✓					0.906 (0.016)	0.917 (0.008)	0.918 (0.011)	0.920 (0.008)	0.987 (0.001)	1.956 (0.642)
		✓				0.892 (0.013)	0.910 (0.009)	0.912 (0.010)	0.905 (0.008)	0.986 (0.001)	2.536 (0.874)
✓			✓			0.918 (0.012)	0.931 (0.008)	0.934 (0.009)	0.935 (0.007)	0.988 (0.001)	1.984 (0.732)
✓			✓	✓		0.914 (0.011)	0.928 (0.008)	0.930 (0.009)	0.932 (0.007)	0.988 (0.001)	2.103 (0.768)
✓			✓		✓	0.928 (0.012)	0.939 (0.008)	0.941 (0.007)	0.943 (0.006)	0.999 (0.000)	1.485 (0.522)

a ECA: Efficient Channel Attention.

b CBAM: Convolutional Block Attention Module.

c GAM: Global Attention Mechanism.

d IoU: intersection over union.

e HD95: 95% Hausdorff distance.

f Component included in the model configuration.

From the table, it can be observed that the lightweight backbone network MobileNetV2 offers advantages in model compactness and parameter efficiency while maintaining competitive accuracy. Its accuracy and robustness are slightly superior to the other backbone networks. Specifically, the IoU coefficient of MobileNetV2 is 0.4% and 1.8% higher than that of Xception and ResNet101, respectively, while its Dice coefficient exceeds those of Xception and ResNet101 by 0.5% and 1.2%, respectively. Building upon this foundation, the incorporation of the GAM further enhances model performance. After introducing GAM, the Dice coefficient and IoU improved significantly, increasing by 0.8% and 1.0%, respectively. Moreover, compared to the Convolutional Block Attention Module (CBAM) integrated at the same position, GAM achieves an additional 1.4% increase in IoU and 1.1% in the Dice coefficient. These results indicate that GAM is more effective in feature extraction and weight allocation, enabling the model to better focus on key information within the kidney tumor region. Furthermore, a comparative analysis of the evaluation metrics in the table reveals that the GAM module not only maintains a balance between precision and recall but also preserves overall segmentation accuracy. This fully validates the effectiveness and generalization capability of the GAM in kidney tumor segmentation tasks.

## Discussion

### Principal Findings

This study introduced GAM-DeepLabV3+, a lightweight, automated framework for renal tumor segmentation, and validated its efficacy on both a single-center clinical dataset and the multicenter KiTS19 benchmark. Our primary finding is that the proposed model achieves superior segmentation precision while maintaining high computational efficiency. On the private dataset, the model yielded a mean DSC of 0.939 (SD 0.008) and a mean HD95 of 1.485 (SD 0.522) pixels, consistently outperforming baseline architectures such as the standard DeepLabV3+ (DSC: mean 0.911, SD 0.008). The robust performance on the KiTS19 external validation set (DSC: mean 0.928, SD 0.006; HD95: mean 2.536, SD 0.742 pixels) further demonstrates the framework’s strong generalizability across heterogeneous data acquisition protocols and varying annotation standards.

Ablation analysis systematically quantified the contributions of the individual architectural innovations (Table 4). Transitioning from an Xception to an enhanced MobileNetV2 backbone provided a 0.5% increase in DSC (from 0.917 to 0.922), confirming that a lightweight backbone can preserve, and even refine, feature representation while significantly reducing computational complexity. Most notably, the substitution of CBAM with the GAM resulted in the most substantial gains, improving the DSC by 1.1% over the CBAM-integrated version. This suggests that the sequential channel-spatial attention design of GAM contributes to suppressing background noise and refining boundary delineation; however, because our evaluation did not include a tumor size–stratified analysis, a specific benefit for small renal lesions remains to be confirmed quantitatively. Furthermore, the reduction in per-slice inference latency (from 21.8 to 14.2 ms/slice on the private dataset, an approximately 1.5× speed-up) indicates improved computational efficiency, although real-time clinical use would require further validation.

### Comparison With Prior Work

The performance metrics in Table 5 illustrate the advantages of the proposed approach relative to previously reported methods on the KiTS19 dataset. Early CNN-based approaches provided a foundation for automated renal tumor segmentation but faced inherent limitations in handling morphological complexity. Yang et al [42] achieved a Dice of 82.6% using a CNN architecture with a 3-stage augmentation strategy; while this result demonstrated the promise of deep learning for this task, the approach encountered difficulties with structurally complex tumor presentations. da Cruz et al [43] advanced the field by introducing a 2.5D DeepLabV3+ model with cross-plane feature fusion, reaching a Dice of 85.17%. Ji et al [44] introduced ASD-Net, a U-Net variant incorporating asymmetric spatial-channel convolution modules, achieving a Dice of 85.22%. In the same period, Ruan et al [45] proposed MB-FSGAN, a multiscale bipath generative adversarial network that reached 85.9%, although its feature extraction remained less sensitive to small lesions.

**Table 5.** Comparison of segmentation metrics of the proposed method versus other state-of-the-art models (KiTS19)^a^.

Work	Methods	Dice	Accuracy
Yang et al [42]	CNNs^b^	0.826	—^c^
da Cruz et al [43]	DeepLabv3+2.5D	0.852	0.997
Ji et al [44]	ASD-Net	0.852	—
Ruan et al [45]	MB-FSGAN	0.859	0.957
Türk et al [46]	Hybrid V-Net	0.865	—
Sun et al [47]	2.5D MFFAU-Net	0.875	—
Jackson et al [48]	3D CNNs	0.885	—
Sun et al [49]	FR2PAttU-Net	0.911	—
This paper	GAM-DeepLabV3+	0.928	0.992

a External Dice values are tumor-class scores from the original 3-class KiTS19 task (background, kidney, and tumor); the proposed method uses a binary tumor-versus-background task with the kidney merged into the background, so the comparison is indicative rather than strictly controlled.

b CNN: convolutional neural network.

c Component not included in the model configuration.

The growing interest in 3D architectures subsequently opened new directions for performance improvement. Türk et al [46] combined residual connections with dense block structures in a hybrid V-Net, achieving a Dice of 86.5%; however, this came at the cost of substantially increased model parameters and computational overhead. Sun et al [47] balanced computational cost against accuracy through a multiscale feature fusion strategy in their 2.5D MFFAU-Net, reaching 87.5%. Jackson et al [48] demonstrated that a 3D CNN augmented with data augmentation could attain a Dice of 88.5%, though the shallow architecture constrained the model’s capacity to represent heterogeneous small-tumor features. More recently, attention mechanisms have driven further performance gains: Sun et al [49] proposed FR2PAttU-Net with a dual-path feature refinement module and pyramid attention unit, achieving a Dice of 91.1%, albeit with a 32% increase in parameter count attributable to its cascaded module design. Notably, the external scores in Table 5 are tumor-class Dice from the original 3-class KiTS19 task (background, kidney, and tumor), whereas our model adopts a binary tumor-versus-background formulation with the kidney merged into the background; the comparison is thus indicative rather than strictly controlled. Moreover, merging the kidney into the background does not necessarily simplify tumor delineation, as the renal parenchyma is the tissue most readily confused with tumor.

The proposed GAM-DeepLabV3+ framework achieved a Dice of 92.8% on the KiTS19 test set, which is numerically higher than the 91.1% Dice reported for FR2PAttU-Net [49], while maintaining an accuracy of 99.2% and a more favorable computational profile. As noted in the footnote to Table 5, this comparison is indicative rather than a strictly controlled head-to-head result, because the external KiTS19 scores derive from the original 3-class task whereas our model adopts a binary tumor-versus-background formulation. On the private dataset, GAM-DeepLabV3+ outperformed both ASD-Net (Dice: 0.876, IoU: 0.810) and DeepLabV3+2.5D (Dice: 0.888, IoU: 0.806), achieving a mean Dice of 0.939 (SD 0.008) and a mean IoU of 0.928 (SD 0.012; Table 6). These results collectively support the effectiveness of combining global context modeling through the GAM module with a lightweight MobileNetV2 encoder for renal tumor segmentation under both data-rich and data-limited conditions. We note that these 2 comparators were reimplemented in-house and evaluated as single-run reference comparisons on the private test set, rather than under the multiseed protocol used for the other baselines in Table 1; they are therefore reported as single-point values.

**Table 6.** Comparison of segmentation metrics of the proposed method versus other state-of-the-art models (private dataset)^a^.

Methods	IoU^b^	Dice	Recall	Precision	Accuracy	HD95^c^
ASD-Net	0.810	0.876	0.869	0.924	0.998	—^d^
DeepLabV3+ 2.5D	0.806	0.888	0.869	0.920	0.998	—
GAM-DeepLabV3+, mean (SD)	0.928 (0.012)	0.939 (0.008)	0.941 (0.007)	0.943 (0.006)	0.999 (0.000)	1.485 (0.522)

a ASD-Net (Ji et al [44]) and DeepLabV3+ 2.5D were reimplemented by us and evaluated on the private test set as single-run reference comparisons; they were not run under the multiseed protocol used for the Table 1 baselines and are therefore reported as single-point values without SDs or HD95. The ASD-Net predictions shown in Figure 7 were produced by this reimplementation; the same reimplementation was also applied to the KiTS19 slices displayed in Figure 7 for qualitative comparison, whereas the quantitative ASD-Net results in this table correspond to the private test set only.

b IoU: intersection over union.

c HD95: 95% Hausdorff distance.

d Not available.

**Figure 8.**
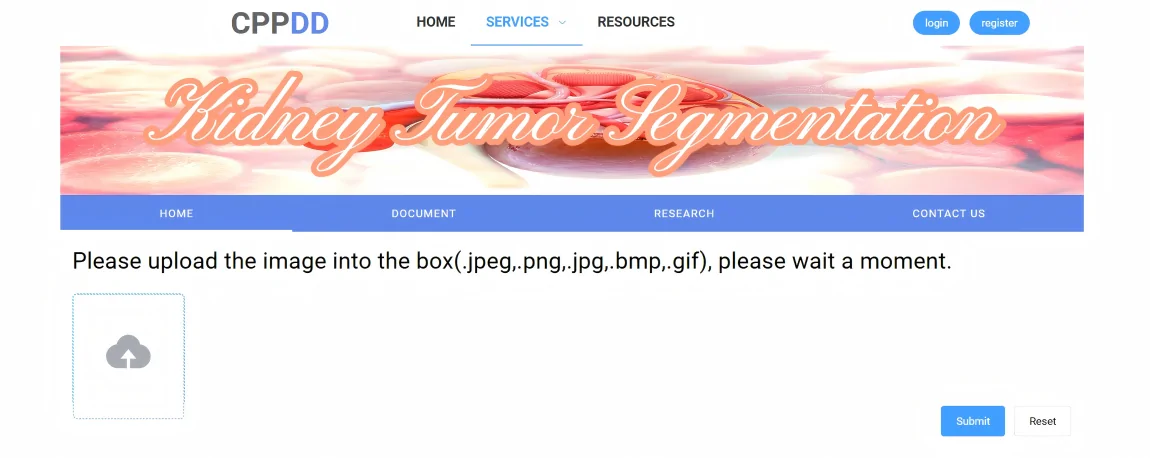
Homepage of the renal tumor segmentation network server.

An online demonstration platform based on GAM-DeepLabV3+ has been made freely accessible at CPPDD [50] (for demonstration purposes only; not intended for upload of real patient data), as illustrated in Figure 8. The platform is intended for research-context demonstration only and is not validated for clinical use.

### Limitations

This study has several limitations that warrant consideration. The private dataset comprised 218 patients from a single center, with one representative 2D slice extracted per patient. Although this strategy is consistent with the 2D segmentation paradigm adopted here, it discards volumetric continuity information and may not fully reflect the spatial heterogeneity of renal tumors across contiguous axial slices. The relatively high performance metrics observed on the private test set may in part reflect the representative nature of the selected slices rather than the full spectrum of imaging variability encountered in clinical practice. To partially address this, external validation was performed on the KiTS19 dataset, which includes multiple consecutive slices per patient and data from multiple centers, providing a more challenging and diverse evaluation environment. The model achieved a mean HD95 of 1.485 (SD 0.522) pixels on the private test set and a mean HD95 of 2.536 (SD 0.742) pixels on KiTS19, suggesting reasonable spatial consistency of the 2D predictions; nevertheless, formal 3D reconstruction validation—including volumetric Surface Dice and 3D HD95 computed across the full *z*-axis stack—to confirm the absence of stacking artifacts remains an important direction for future investigation. This constitutes a key limitation of the current work. In addition, because the external KiTS19 results in Table 5 were obtained under different task definitions (3-class vs binary), preprocessing, and evaluation protocols, the numerical differences reflect contextual competitiveness rather than a controlled demonstration of superiority. Finally, although the architecture is motivated in part by the difficulty of small renal tumors, no tumor size–stratified evaluation was performed; the corresponding claims are therefore qualitative, and a volume-stratified analysis of small-lesion performance is left for future work. Furthermore, because only 2D slices containing annotated renal tumors were retained and tumor-free (negative) slices were discarded during dataset construction, the model’s false-positive behavior on normal, tumor-free tissue and its performance on complete patient volumes that include negative slices were not evaluated in this study; assessment on full CT volumes therefore remains an important direction for future work.

Although nnU-Net was included as a baseline, only its 2D implementation was evaluated under our unified experimental protocol. This baseline therefore represents a custom adaptation that may underestimate the true capability of the nnU-Net framework. The complete self-configuring nnU-Net pipeline and whole-volume 3D evaluation were not explored, which should be addressed in future work. Likewise, modern ViT baselines were not included in our comparative experiments, and a direct experimental comparison against Transformer-based architectures remains future work. In addition, because of the retrospective design and limited dataset scale, the reported results should be interpreted as indicating promising accuracy for research and assistive purposes rather than as evidence of validated clinical utility. The private test set included only 22 patients, which may limit the statistical power to detect significant performance differences between methods. The heterogeneous nature of renal tumors across stages and histological subtypes, combined with the single-institution data source, may limit the generalizability of the current findings. Prospective multicenter studies with larger and more diverse patient cohorts are needed before conclusions regarding clinical deployment can be made with confidence.

### Conclusions

In summary, this study proposed GAM-DeepLabV3+, a CT renal tumor segmentation model built on an improved DeepLabV3+ framework incorporating a lightweight MobileNetV2 encoder, an ASPP module, and a GAM attention mechanism in the decoder. The model demonstrated promising segmentation accuracy on both the private dataset (Dice: mean 0.939, SD 0.008; IoU: mean 0.928, SD 0.012; HD95: mean 1.485, SD 0.522 pixels) and the public KiTS19 dataset (Dice: mean 0.928, SD 0.006; IoU: mean 0.902, SD 0.008; HD95: mean 2.536, SD 0.742 pixels), while offering improved inference speed relative to standard DeepLabV3+. By integrating lightweight backbone design with global-local attention feature fusion, the proposed approach is designed to address several challenges in renal tumor CT segmentation, such as blurred tumor boundaries and class imbalance; a dedicated, tumor size–stratified evaluation of small-lesion performance remains an avenue for future work. These findings support the potential of GAM-DeepLabV3+ as a research tool and a basis for further development toward clinically assistive automated segmentation systems. Prospective validation on larger, multicenter datasets will be an essential next step in assessing broader clinical applicability.

## Abbreviations

ASPP: Atrous Spatial Pyramid Pooling
BAU-Net: boundary attention U-Net
BCE: binary cross-entropy
CBAM: Convolutional Block Attention Module
ccRCC: clear cell renal cell carcinoma
chRCC: chromophobe renal cell carcinoma
CNN: convolutional neural network
Conv1D: 1D convolution
CRF: conditional random field
CT: computed tomography
DSC: Dice similarity coefficient
Dw Conv: depthwise convolution
ECA: Efficient Channel Attention
FN: false negative
FP: false positive
FPN: feature pyramid network
FReLU: free rectified linear unit
GAM: Global Attention Mechanism
GAP: global average pooling
GPU: graphics processing unit
HD95: 95% Hausdorff distance
IoU: intersection over union
MLP: multilayer perceptron
MSS U-Net: multiscale supervised U-Net
NIfTI: Neuroimaging Informatics Technology Initiative
nnU-Net: no-new-Net
pRCC: papillary renal cell carcinoma
RAU-Net: residual and attention U-Net
ReLU: rectified linear unit
TN: true negative
TP: true positive
VHL: von Hippel-Lindau
ViT: Vision Transformer
WHO: World Health Organization
WL: window level
WW: window width

## References

[1] Baniak NJ (2025). Differential diagnosis of renal neoplasia with clear cell cytology. Kidney Cancer.

[2] Paner G, Cimadamore A, Franzese C (2025). Oncocytic tumors in the kidney: a trifocal review - integrated pathological, cytopathological, and molecular perspectives (Part 1). Acta Cytol.

[3] Vasantbhai Patel V, Yadav AR, Jain P, Cenkeramaddi LR (2024). A systematic kidney tumour segmentation and classification framework using adaptive and attentive-based deep learning networks with improved crayfish optimization algorithm. IEEE Access.

[4] Kumar Y, Brar TPS, Kaur C, Singh C (2024). A comprehensive study of deep learning methods for kidney tumor, cyst, and stone diagnostics and detection using CT images. Arch Computat Methods Eng.

[5] Alaghehbandan R, Siadat F, Trpkov K (2023). What’s new in the WHO 2022 classification of kidney tumours?. Pathologica.

[6] Checcucci E, De Cillis S, Granato S (2020). Applications of neural networks in urology: a systematic review. Curr Opin Urol.

[7] Xie Y, Ma X, Li H (2017). Prognostic value of clinical and pathological features in Chinese patients with chromophobe renal cell carcinoma: a 10-year single-center study. J Cancer.

[8] Sadaghiani MS, Baskaran S, Gorin MA (2024). Utility of PSMA PET/CT in staging and restaging of renal cell carcinoma: a systematic review and metaanalysis. J Nucl Med.

[9] Bi WL, Hosny A, Schabath MB (2019). Artificial intelligence in cancer imaging: clinical challenges and applications. CA Cancer J Clin.

[10] Rowe SP, Pomper MG (2022). Molecular imaging in oncology: current impact and future directions. CA Cancer J Clin.

[11] Liu P, Chen G, Sang Z, Niu Y (2025). The role of three-dimensional reconstruction in partial nephrectomy for ipsilateral multifocal renal tumors: a multicenter retrospective study. BMC Urol.

[12] Yu Q, Shi Y, Sun J, Gao Y, Zhu J, Dai Y (2019). Crossbar-Net: a novel convolutional neural network for kidney tumor segmentation in CT images. IEEE Trans Image Process.

[13] Myronenko A, Hatamizadeh A (2019). 3D kidneys and kidney tumor semantic segmentation using boundary-aware networks. arXiv.

[14] Heller N, McSweeney S, Peterson MT (2020). An international challenge to use artificial intelligence to define the state-of-the-art in kidney and kidney tumor segmentation in CT imaging. J Clin Oncol.

[15] Zhao W, Jiang D, Peña Queralta J, Westerlund T (2020). MSS U-Net: 3D segmentation of kidneys and tumors from CT images with a multi-scale supervised U-Net. Inform Med Unlocked.

[16] Guo J, Zeng W, Yu S, Xiao J RAU-net: u-net model based on residual and attention for kidney and kidney tumor segmentation. https://ieeexplore.ieee.org/xpl/mostRecentIssue.jsp?punumber=9341295.

[17] Zhao Z, Chen H, Li J, Wang L (2022). Boundary attention U-Net for kidney and kidney tumor segmentation. Annu Int Conf Eng Med Biol Soc.

[18] Rao PK, Chatterjee S, Janardhan M (2023). Optimizing inference distribution for efficient kidney tumor segmentation using a UNet-PWP deep-learning model with XAI on CT scan images. Diagnostics (Basel).

[19] Ranjbarzadeh R, Keleş A, Crane M (December 3, 2024). Ebtag: explainable brain tumor segmentation with efficientnetv2, attention mechanism, and grad-cam in clinical and public datasets. SSRN.

[20] Safarpour H, Sadeghi S, Zarbakhsh P, Kamsari M, Kia M, Ranjbarzadeh R (2026). Explainable deep learning framework for brain tumor segmentation using vision transformer and conditional random fields. Multimedia Systems.

[21] Choi SR, Ko K, Baek SJ, Lee S, Lee J, Lee M Enhanced kidney tumor segmentation in CT scans using a simplified UNETR with organ information.

[22] Abdelrahman A, Viriri S (2022). Kidney tumor semantic segmentation using deep learning: a survey of state-of-the-art. J Imaging.

[23] Ranjbarzadeh R, Anari S, Crane M, Bendechache M A hybrid UNet and vision transformer architecture with multi-scale fusion for brain tumor segmentation.

[24] Safarpour H, Anari S, Ranjbarzadeh R, Safavi S, Bendechache M (July 25, 2025). A dual-phase segmentation framework utilizing gumbel-softmax and a cascaded swin transformer for multi-class brain tumor segmentation. In Review.

[25] Ranjbarzadeh R, Crane M, Bendechache M (2025). The impact of backbone selection in YOLOv8 models on brain tumor localization. Iran J Comput Sci.

[26] Ranjbarzadeh R, Keles A, Ozisik PA Combining DeepLabV3 with attention mechanisms for accurate brain tumor segmentation: insights from BraTS 2020 and a private clinical dataset.

[27] Kan HC, Fan GM, Wei MH (2025). Automated kidney tumor segmentation in CT images using deep learning: a multi-stage approach. Acad Radiol.

[28] Swapna J, Kiruba RR (2026). Deep learning based segmentation of renal cancer detection and segmentation enhanced 3D CT images. J Electr Eng Technol.

[29] Lin Z, Cui Y, Liu J (2021). Automated segmentation of kidney and renal mass and automated detection of renal mass in CT urography using 3D U-Net-based deep convolutional neural network. Eur Radiol.

[30] Gido M, Nakagawa S, Mori K, Kakeya H (2025). [Paper] Kidney and renal tumor segmentation by nnU-Net Using 3D CT data from different sources. ITE Trans Media Technol Appl.

[31] Vaswani A, Shazeer N, Parmar N, Guyon I, von Luxburg U, Bengio S (2017). Advances in Neural Information Processing Systems 30 Proceedings of the 31st Annual Conference on Neural Information Processing Systems.

[32] Karunanayake N, Lu L, Yang H (2025). Dual-stage AI model for enhanced CT imaging: precision segmentation of kidney and tumors. Tomography.

[33] Heller N, Isensee F, Maier-Hein KH (2021). The state of the art in kidney and kidney tumor segmentation in contrast-enhanced CT imaging: results of the KiTS19 challenge. Med Image Anal.

[34] KiTS19. GitHub.

[35] Li J, Liu K, Hu Y (2023). Eres-UNet++: liver CT image segmentation based on high-efficiency channel attention and Res-UNet++. Comput Biol Med.

[36] Chen LC, Zhu Y, Papandreou G, Schroff F, Adam H, Ferrari V, Hebert M, Sminchisescu C, Weiss Y Computer Vision – ECCV 2018 Lecture Notes in Computer Science.

[37] Chollet F Xception: deep learning with depthwise separable convolutions.

[38] Sandler M, Howard A, Zhu M, Zhmoginov A, Chen LC MobileNetV2: inverted residuals and linear bottlenecks.

[39] Chen LC, Papandreou G, Kokkinos I, Murphy K, Yuille AL (2018). DeepLab: semantic image segmentation with deep convolutional nets, atrous convolution, and fully connected CRFs. IEEE Trans Pattern Anal Mach Intell.

[40] Liu Y, Shao Z, Hoffmann N (December 10 2021). Global attention mechanism: retain information to enhance channel-spatial interactions. arXiv.

[41] Wang Q, Wu B, Zhu P, Li P, Zuo W, Hu Q ECA-net: efficient channel attention for deep convolutional neural networks. https://ieeexplore.ieee.org/xpl/mostRecentIssue.jsp?punumber=9142308.

[42] Yang G, Wang C, Yang J (2020). Weakly-supervised convolutional neural networks of renal tumor segmentation in abdominal CTA images. BMC Med Imaging.

[43] da Cruz LB, Júnior DAD, Diniz JOB (2022). Kidney tumor segmentation from computed tomography images using DeepLabv3+ 2.5D model. Expert Syst Appl.

[44] Ji Z, Mu J, Liu J (2024). ASD-Net: a novel U-Net based asymmetric spatial-channel convolution network for precise kidney and kidney tumor image segmentation. Med Biol Eng Comput.

[45] Ruan Y, Li D, Marshall H (2020). MB-FSGAN: Joint segmentation and quantification of kidney tumor on CT by the multi-branch feature sharing generative adversarial network. Med Image Anal.

[46] Türk F, Lüy M, Barışçı N (2020). Kidney and renal tumor segmentation using a hybrid V-Net-based model. Mathematics.

[47] Sun P, Mo Z, Hu F (2023). 2.5D MFFAU-Net: a convolutional neural network for kidney segmentation. BMC Med Inform Decis Mak.

[48] Jackson P, Hardcastle N, Dawe N, Kron T, Hofman MS, Hicks RJ (2018). Deep learning renal segmentation for fully automated radiation dose estimation in unsealed source therapy. Front Oncol.

[49] Sun P, Mo Z, Hu F (2022). Kidney tumor segmentation based on FR2PAttU-Net model. Front Oncol.

[50] KAI. Central Platform of Digital Disease (CPPDD).

[51] GAM-deeplabv3plus-kidneytumor. GitHUb.

